# Effects of Sodium–Glucose Co-Transporter 2 Inhibitors on Serum Chloride Concentrations in Patients with Heart Failure

**DOI:** 10.3390/jcdd11110364

**Published:** 2024-11-09

**Authors:** Ivana Jurin, Vanja Ivanović Mihajlović, Zrinka Šakić, Marin Pavlov, Tomislav Šipić, Petra Vitlov, Hrvoje Falak, Danijela Grizelj, Šime Manola, Mario Udovičić

**Affiliations:** 1 Department for Cardiovascular Diseases, Dubrava University Hospital, 10000 Zagreb, Croatia; ivanajurin1912@gmail.com (I.J.); marin.pavlov@gmail.com (M.P.); tomislavsipic2@gmail.com (T.Š.); petra.vitlov@gmail.com (P.V.); hrvoje.falak@gmail.com (H.F.); danijela.grizelj@yahoo.com (D.G.); sime.manola@icloud.com (Š.M.); mario.udovicic@gmail.com (M.U.); 2 Vuk Vrhovac University Clinic for Diabetes, Endocrinology and Metabolic Diseases, Merkur University Hospital, 10000 Zagreb, Croatia; sakiczrinka@gmail.com; 3 Professional Undergraduate Study Physiotherapy, University North, 48000 Koprivnica, Croatia; 4 School of Medicine, Catholic University of Croatia, 10000 Zagreb, Croatia; 5 School of Medicine, University of Zagreb, 10000 Zagreb, Croatia

**Keywords:** heart failure, sodium–glucose co-transporter 2 inhibitors, chloride, Na/Cl ratio

## Abstract

Background and aims: In the past few years, some reports have shown that serum chloride concentration is a more powerful prognostic predictor than serum sodium levels in heart failure (HF). Elevated Na/Cl ratio has shown to be independently associated with all-cause death in acute HF. We evaluated changes in serum chloride concentrations and Na/Cl ratio in correlation with various clinical factors during 12 months of follow-up in patients in whom SGLT2is were initiated as part of HF therapy. Patients and methods: This was a prospective observational study conducted at University Hospital Dubrava and involving patients with HF. We included 241 participants between May 2021 and April 2023. All data were obtained before the introduction of SGLT2is, and the same parameters were obtained at 6 and 12 months of follow-up as well. Results: The results show that higher chloride concentration at both 6 and 12 months is an independent predictor of lower NT-proBNP levels. The chloride concentrations did not differ significantly between these groups in the follow-up period. There were no statistically significant differences in the Na/Cl ratio at different timepoints. The presence of cardiovascular risk factors did not significantly affect the increase in chloride concentration. Conclusions: Our results suggest that hypochloremia could be a potentially modifiable risk factor, given the influence of SGLT2is on chloride concentration, but also an ominous sign of a poor outcome in patients with HF. We believe that the determination of chloride concentrations should become routine in the monitoring of patients with HF.

## 1. Introduction

Pathophysiological mechanisms that are present in patients with heart failure (HF) often cause abnormalities in electrolyte concentrations. Those electrolyte abnormalities are the result of a synergistic effect not only of pathophysiological mechanisms that include neurohumoral activation but also of renal impairment and diuretic therapy [1]. Imbalances in the serum concentrations of potassium and sodium were historically of great clinical interest, since they may require tailoring of guideline-directed medical therapy (GDMT) and, therefore, appear to be associated with prognosis [2]. Although chloride and sodium are very potent anions in the extracellular fluid, chloride has been unfairly neglected not only in clinical trials but in everyday practice as well, because clinicians saw its role only as a passive one in maintaining electrical neutrality [3]. A possible reason for this unfair neglect of chloride throughout history lies in the fact that several studies have suggested that low sodium concentrations are one of the most powerful predictors of short- and long-term morbidity and mortality in HF, irrespective of left ventricular systolic function [4,5,6].

In 2015, Grodin et al. [7] reported that low serum chloride concentrations on admission were independently associated with an increased risk of mortality in patients with acute HF. In their next publication, they investigated the effect of serum chloride in patients with chronic HF and found that for every 4.1 mmol/L decrement in serum chloride concentration, there was a 26–29% increase in 5-year mortality risk [8]. After those reports, serum chloride, until that time a “neglected electrolyte”, started to gain more attention, especially in the field of HF pathophysiology, leading to “chloride theory” [9]. “Chloride theory” has put chloride at the center of the body fluid status. According to “chloride theory”, two distinct modes of worsening HF progression are hypothesized, with different clinical characteristics according to the serum chloride concentration—one in which serum chloride concentrations are increased, and one in which serum chloride concentrations are decreased [9,10,11]. Hypochloremia has been identified as a clinically significant prognostic marker after several studies have shown its association with neurohumoral activation, diuretic resistance, and a worse prognosis in patients with HF [12,13,14]. Hypochloremia is frequently encountered in patients with HF and is commonly associated with the use of loop and thiazide diuretics, which also cause metabolic alkalosis, which decreases respiratory activity, resulting in a possible vicious cycle of worsening HF [15]. To date, there are no guideline-directed therapies for hypochloremia, and only possible solutions have been postulated [16]. One of the proposed solutions is that treating hypochloremia with chloride-regaining diuretics such as acetazolamide or sodium–glucose co-transporter 2 inhibitors (SGLT2is) might correct the metabolic alkalosis and, therefore, correct the vicious cycle of worsening HF [16,17,18,19]. Based on our previous knowledge, both hyponatremia and hypochloremia are associated with worse prognosis in patients with HF. SGLT2is interfere with the reabsorption of glucose and sodium in the early proximal renal tubule and, therefore, can prevent hyponatremia via osmotic diuresis [20].

In a recent study, Fernando et al. [21] investigated the prognostic significance of the sodium-to-chloride (Na/Cl) ratio in patients experiencing acute HF and found that elevated Na/Cl ratio was independently associated with all-cause death. However, since this was a retrospective study, these patients did not have SGLT2is as a part of their GDMT therapy. The results of this study emphasized the importance of monitoring of sodium and chloride concentrations, as correcting electrolyte abnormalities may markedly enhance patient survival rates [21,22].

Our knowledge of the action of SGLT2is is growing day by day; apart from the possible correction of electrolyte imbalance that can improve outcomes, it is probably all due to the synergistic effect of not only the correction of that electrolyte imbalance but also the positive effect on left ventricular remodeling [23]. However, to the best of our knowledge, changes in serum chloride concentrations and sodium-to-chloride ratio, along with their associations with N-terminal fragment of pro-brain natriuretic peptide (NT-proBNP) concentration during 6 and 12 months of follow-up in patients in whom SGLT2is are initiated as part of HF therapy, and their impact on prognosis, are unknown. Therefore, the aim of this study was to evaluate those relations.

## 2. Materials and Methods

### 2.1. Study Design and Setting

This was a prospective observational study conducted at University Hospital Dubrava and involving patients with HF. Patients were recruited from the local HF registry, the CaRD registry (NCT06090591). In brief, this registry includes patients with HF in whom de novo therapy with SGLT2is has been commenced, regardless of HF aetiology (non-ischemic/ischemic) or setting (acute, de novo, acute-on-chronic; hospitalized, outpatient clinic visit). Patients were treated with GDMT, and HF diagnosis and phenotyping were established according to the updated guidelines of the European Society of Cardiology [24].

### 2.2. Inclusion and Exclusion Criteria

Inclusion criteria were the diagnosis of heart failure, regardless of left ventricular ejection fraction, and age over 18 years. We included patients with preserved (HFpEF—patients with left ventricular ejection fraction ≥ 50%), mildly reduced (HFmrEF—patients with left ventricular ejection fraction between 41 and 49%), and reduced ejection fraction (HFrEF—left ventricular ejection fraction ≤ 40%) in our study, according to the guidelines [24]. We excluded those patients for whom we did not have adequate data and those who had contraindications or obstacles to the introduction of SGLT2is (patients with diabetes mellitus type 1, patients with eGFR < 25 mL/min, and pregnant women). Patients in whom SGLT2i therapy was terminated were not excluded from the registry, due to the small number of patients (5%), who all terminated the therapy after 6 months of follow-up.

### 2.3. Data Collection

Overall, we included 241 participants between May 2021 and April 2023. We collected the patients’ sociodemographic data, body mass index, heart rate, NYHA status, comorbidities, previous therapy data, smoking status, and laboratory values. Hyponatremia was considered when the serum sodium concentration was below 137 mmol/L, and hypochloremia was defined as a serum chloride concentration < 96 mmol/L, following the laboratory reference limits. Sodium and chloride concentrations were measured using conventional methods with a Beckman–Coulter^®^ AU5400^®^ automated clinical chemistry analyzer (Brea, CA, USA). NT-proBNP serum concentrations were obtained using Alinity i (Abbott Laboratories, Green Oaks, IL, USA). The Na/Cl ratio formula was serum sodium divided by serum chloride. All data were obtained before the introduction of SGLT2is, and the same parameters were obtained at 6 and 12 months of follow-up as well.

### 2.4. Outcome Measures

The primary outcome was changes in chloride concentration during the follow-up time. The secondary outcomes were the correlations between chloride concentrations and NT-proBNP concentrations, chloride concentrations and functional status of the patients, as well as correlation between Na/Cl ratio and NT-proBNP concentrations. Additionally, the secondary outcome was to investigate the interdependance between NT-pro BNP concentrations and other patient-specific parameters.

### 2.5. Ethical Considerations

This study has been approved by the University Hospital Dubrava Ethics Committee (2022/1403-01).

### 2.6. Statistical Analysis

Data are presented as medians with interquartile ranges (IQRs) for continuous variables, and as counts with proportions for categorical variables. The normality of the data was checked using the Shapiro–Wilk test. Continuous data were analyzed using the Kruskal–Wallis test with the post hoc Mann–Whitney U test. Non-parametric repeated-measures ANOVA (Friedman test) was used for longitudinal data (repeatedly measured from the same patient). Categorical data were analyzed using the χ^2^ test. The association of different covariates was tested with a multivariable linear regression model for continuous outcomes, and with logistic regression for categorical outcomes. Binary logistic regression was used for binary outcomes, while ordinal logistic regression was used for ordinal outcomes. A *p*-value less than 0.05 was considered statistically significant. Data were collected and prepared using Microsoft Excel and statistically analyzed using Jamovi (The jamovi project (2024); jamovi (Version 2.3.16.0) [Computer Software]. Retrieved from https://www.jamovi.org, Sydney, Australia, accessed on 6 June 2024.

## 3. Results

### 3.1. Participants’ Demographic and Clinical Characteristics, Including Therapy

This was a prospective observational study and included 241 patients. Baseline demographic characteristics of the study population are shown in Table 1.

Table 2 shows concomitant therapy in participants before the introduction of SGLT2 inhibitors.

**Figure 1:**
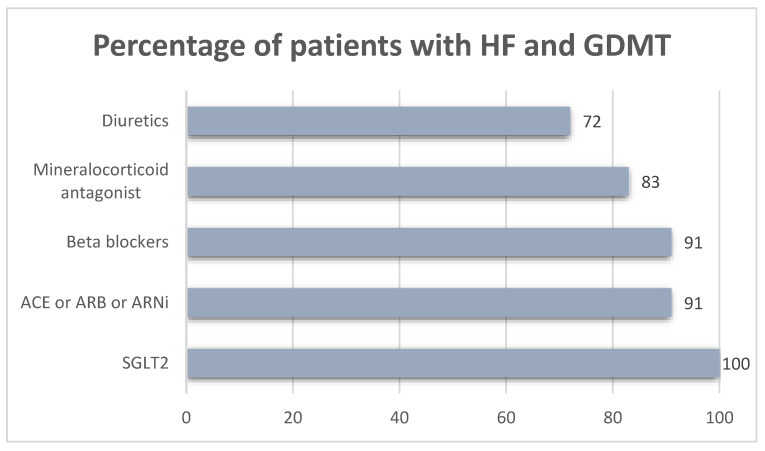
Percentage of patients with introduced guideline-based medications.

Figure 1 shows the percentage of patients with introduced guideline based medical therapy, before the introduction of SGLT2 inhibitors.

### 3.2. Chloride Concentrations and Na/Cl Ratios at Different Timepoints

Table 3 shows chloride concentrations at baseline, 6 months and at 12 months of follow up.

**Figure 2:**
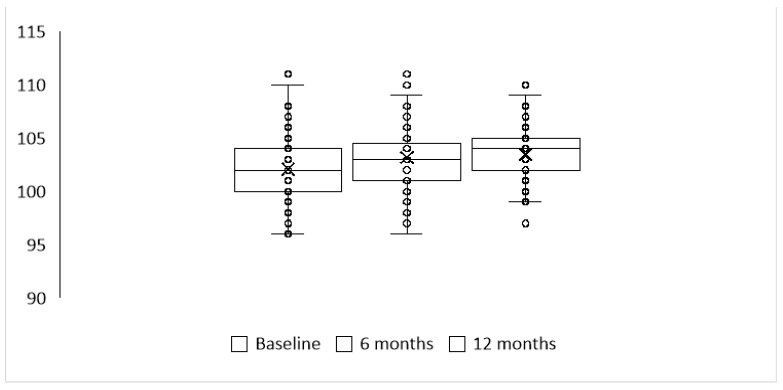
Chloride concentration at baseline, 6 months, and 12 months. The mean, median and range of chloride concentrations at the baseline, 6 months and 12 months of follow-up. The ranges are shown as dots, interquartile ranges as boxes, median and mean values are shown as solid lines and crosses, respectively, inside the boxes.

Figure 2 shows graphically the dynamic of chloride concentrations at baseline, 6 months and 12 months of follow up.

Since the chloride concentrations were not normally distributed (Shapiro–Wilk test *p*-values < 0.001 at all timepoints), non-parametric repeated-measures ANOVA (Friedman test) was used to assess changes in chloride concentration over time. A *p*-value < 0.001 indicates that the chloride concentration significantly differed at different timepoints. According to post hoc analysis (Durbin–Conover test with *p*-values adjusted after Bonferroni correction), the difference was significant at 0 vs. 6 months (*p* < 0.001) and 0 vs. 12 months (*p* < 0.001) but was insignificant at 6 vs. 12 months (*p* = 1.000).

Table 4 shows the Na/Cl ratio at baseline, 6 months and 12 months of follow up.

**Figure 3:**
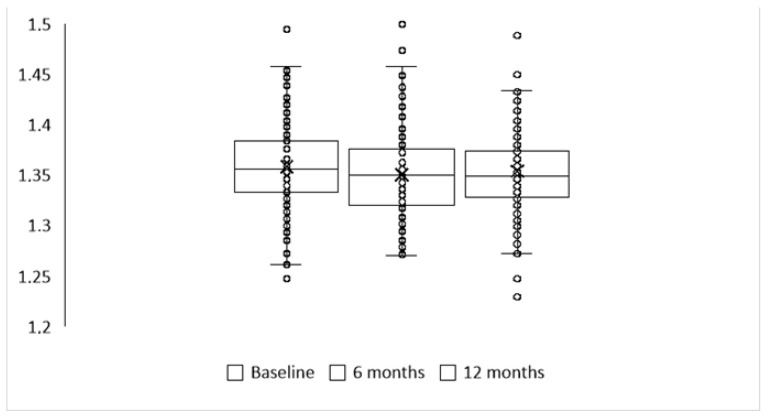
Na/Cl ratio at baseline, 6 months, and 12 months. The mean, median and range of Na/Cl ratios at the baseline, 6 months and 12 months of follow-up. The ranges are shown as dots, interquartile ranges as boxes, median and mean values are shown as solid lines and crosses, respectively, inside the boxes.

Figure 3 shows graphically the dynamic of Na/Cl ratio at baseline, 6 months and 12 months of follow up.

Since the Na/Cl ratio values were not normally distributed (Shapiro–Wilk test *p*-values < 0.05 at all timepoints), non-parametric repeated-measures ANOVA (Friedman test) was used to asses the change in Na/Cl ratio values over time. The obtained *p*-value of 0.137 indicates there were no statistically significant differences in Na/Cl ratio between different timepoints.

### 3.3. Differences Between HF Groups

Table 5 shows how the values of chloride concentrations and Na/Cl ratios during follow-up differed depending on the initial HF group. The results indicate that the HF group did not significantly affect any variable except the Na/Cl ratio at 6 months. Post hoc analysis revealed that the observed significance was due to the statistically significant difference between HFmrEF and HFrEF (*p* = 0.013).

Table 6 shows the dynamics of NT-proBNP values in different HF groups.

**Figure 4:**
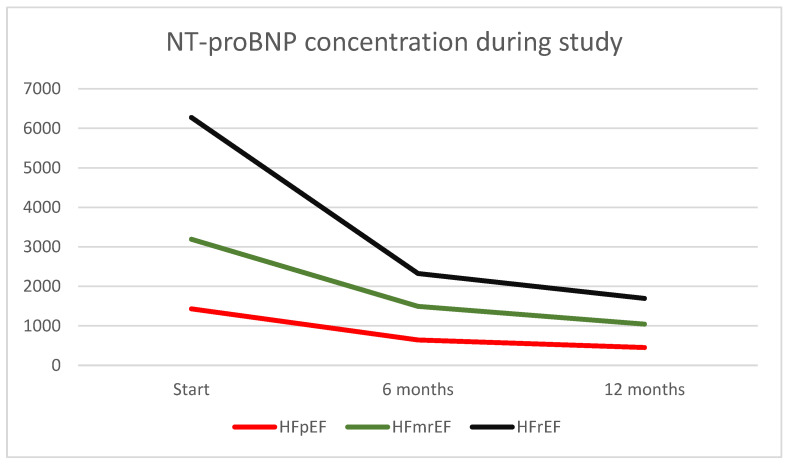
Dynamics of NT-proBNP concentration in the follow-up period between different HF groups.

Figure 4 shows the dynamics of NT-proBNP concentrations at baseline, 6 months and at the 12 months of follow-up.

### 3.4. Relationship Between Chloride Concentration, Na/Cl Ratio, and Level of NT-proBNP

To explore the relationship between chloride concentration, Na/Cl ratio, and level of NT-proBNP, two multivariable linear regression analyses were performed, taking demographic data as covariates. The first analysis included chloride concentration and the second included Na/Cl ratio as covariates. Two separate regression models were designed due to the strong correlation between chloride concentration and Na/Cl ratio at all timepoints (Pearson correlation coefficients were −0.81, −0.73, and −0.83 at baseline, 6 months, and 12 months, respectively). Both analyses included only statistically significant covariates from univariate analysis. Since NT-proBNP was significantly right-skewed, it was logarithmically transformed. The results of the analyses are shown in Table 7 and Table 8.

The results show that higher chloride concentrations at baseline, 6 months, and 12 months are independent predictors of lower NT-proBNP levels, while higher values of Na/Cl ratio are independent predictors of higher NT-proBNP levels at all timepoints. Values of R^2^ indicate that both models similarly predict the value of NT-proBNP.

Furthermore, age positively influenced the NT-proBNP levels at all timepoints. In addition, higher BMI at 6 and 12 months was an independent predictor of lower NT-proBNP levels. While the association of BMI and NT-proBNP at baseline was significant in univariate analysis, it was not significant in multivariate analysis when adjusted for chloride concentration and age. Sex and smoking did not affect NT-proBNP levels.

### 3.5. Effects of Different Patient-Related Factors on Chloride Concentration

In order to explore which cardiovascular risk factors, echocardiographic parameters, laboratory values, and medications affected changes in chloride concentration, multivariate binary logistic regression was performed. An increase in chloride concentration was a binary outcome in the regression model. Multivariate logistic regression included only statistically significant covariates from the univariate model and is shown in Table 9.

### 3.6. Effects of Different Patient-Related Factors on Number of Hospital Admissions Due to Worsening Heart Failure

The effects of cardiovascular risk factors, echocardiographic parameters, and initial laboratory values on the number of hospital admissions due to worsening heart failure during follow-up were tested in a multivariate ordinal logistic regression model that included only statistically significant covariates from univariate analysis and is shown in Table 10. The number of hospital admissions due to worsening heart failure was taken as an ordinal outcome.

The results show that higher initial chloride concentrations were significantly associated with decreased odds of hospital admission. While higher LVEF values significantly corresponded to smaller odds of readmission in univariate analysis, the results were non-significant when adjusted for chloride concentration. All other covariates did not significantly affect the odds of hospital readmission.

### 3.7. Correlation Between Chloride Concentrations and NYHA Functional Class

The median chloride concentrations for different NYHA groups at 6 and 12 months are shown in Table 11. The results show that chloride concentrations significantly differed between NYHA groups at both timepoints.

## 4. Discussion

In our prospective, observational cohort of 241 patients, we evaluated changes in serum chloride concentrations as well as sodium-to-chloride ratio (Na/Cl ratio) and their associations with NT-proBNP concentration during 6 and 12 months of follow-up in patients in whom SGLT2is were initiated as part of HF therapy. To the best of our knowledge, this is the first real-world study to evaluate abovementioned correlations.

After decades of the “reign of sodium” in preserving fluid homeostasis, chloride started to gain more attention when “Chloride theory” was proposed. The “Chloride theory” suggests that chloride is central to regulating fluid homeostasis in HF [9,11]. The macula densa in the juxtaglomerular apparatus is sensitive to chloride, leading to increased renin release and activation of the renin–angiotensin–aldosterone system (RAAS) when chloride concentrations are low [9,22]. Increased renin release affects myocardial conduction and contractility, leading to worsening of HF [22]. Both hyponatremia and hypochloremia are associated with worse prognosis in patients with HF [4,5,6,13,14,25]. SGLT2is are the only medication that has proven to be effective over the entire HF spectrum [24]. Although we still do not know all of the mechanisms by which SGLT2is reduce the burden of HF, we can certainly assume that one of those mechanisms might be the correction of electrolyte imbalance, particularly sodium and chloride. SGLT2is can modulate non-osmotic sodium storage, decreasing the skin’s sodium content and interstitial fluid volume and improving dilutional hyponatremia by increasing erythropoietin production and hematocrit [26]. However, data on the short- and long-term effects of SGLT2 inhibitors on serum sodium concentrations are scarce. Regarding the effects of SGLT2is on increasing serum chloride concentrations, this is probably caused by SGLT2is’ osmotic diuretic action, with subsequent water excretion and mild hemoconcentration [27]. A study by Kataoka et al. [19] demonstrated that the SGLT2i empagliflozin enhances the serum chloride concentration in type 2 diabetes mellitus (T2DM) patients without HF and showed that chloride concentration could be increased by enhanced reabsorption of urinary chloride in proximal tubules, or via the buffering activity of strong organic acid metabolites. One study in Japan evaluated the impact of the SGLT2i tofogliflozin on serum chloride levels over a 3-month period. The study was retrospective in design and included 64 patients aged ≥ 65 years with T2DM, demonstrating a significant increase in serum chloride concentration, mainly in elderly individuals with T2DM who were taking tofogliflozin [28]. In our study, the patients were taking either empagliflozin or dapagliflozin, because those SGLT2is are currently available in our country, and they are also SGLT2is with the IA recommendation and level of evidence for the treatment of heart failure according to the ESC guidelines [24].

We found that SGLT2is significantly increased the chloride concentration at 6 and 12 months of follow-up compared to the initial concentration at the time of introduction. However, the chloride concentrations after 6 and 12 months did not differ significantly. Earlier studies have shown that SGLT2is achieve their clinical effect very quickly. When examining their effects on HFrEF patients, after the 28th day of administration, a statistically significant reduction in the risk of death from cardiovascular causes and in the risk of worsening HF was achieved [29]. In patients with HFpEF, the same effect was noted even earlier, after the 13th day of administration [30]. How quickly SGLT2is affect certain biochemical parameters has not been investigated so far. However, considering the very fast clinical effect, we assume that the effect on biochemical parameters also occurs soon after application, which could be the reason why the chloride values in our study after 6 and 12 months did not differ significantly. Certainly, additional studies will be needed to test our hypothesis. The results of such research would have a potential practical application in monitoring patients and monitoring the effects of therapy, bearing in mind that, as we mentioned earlier, chloride is increasingly recognized as a reliable prognostic marker in heart failure [7,13]. It would apparently be particularly useful in countries where NT-proBNP is not readily available or is expensive to determine. In these situations, chloride concentrations could possibly give a relevant insight into the patient’s condition.

As already stated, for the majority of patients, the impact of the newly introduced SGLT2is manifested in an increase in chloride concentration, even to a significant extent. However, in some patients, the chloride concentration did not increase. We were interested in more details to determine whether other factors, such as comorbidities and some laboratory values of echocardiographic data, influence the increase in chloride concentration. For this purpose, we divided our population of subjects into those with and without increased chloride concentrations. The results showed that the presence of cardiovascular risk factors such as coronary disease and T2DM does not significantly affect the increase in chloride concentration. Also, the initial concentration of NT-proBNP, as well as the initial LVEF, did not significantly affect the chloride concentration changes.

We obtained the same result by comparing groups of patients depending on whether they belonged to the HFrEF, HFmrEF, or HFpEF group. Chloride concentrations did not differ significantly between these groups in the follow-up period. Interpretation of our results represents a great challenge, since the data with which we could compare our results are scarce. Pathophysiologically, HF is certainly a very complex syndrome, independent of LVEF and initial NT-proBNP concentration, with neurohumoral activation as one of the central events in the entire cascade. There are many interactions and pathways that are yet not fully understood, especially taking into account the influence of powerful drugs such as SGLT2is. On the other hand, the possible interaction between chloride concentration and the impact of other drugs, besides SGLT2is, is more predictive, although it has also not been investigated enough.

The ESC guidelines [24] recommend the use of four main groups of drugs in heart failure therapy: Angiotensin-converting enzyme (ACE) inhibitors/angiotensin receptor blockers (ARBs) or angiotensin receptor/neprilysin inhibitor (ARNI), beta-blockers, mineralocorticoid receptor antagonists (MRAs), and SGLT2is for HFrEF. Recently, SGLT2is have become mandatory in patients with HfpEF [24]. Clinical guidelines include diuretics for the treatment of HF as the first-line therapy to prevent and treat fluid overload and to improve symptoms, although there is no evidence that they improve survival in patients with HF. To what extent other GDMTs, besides SGLT2is, affect the chloride concentration is not known. Treatment with MRAs can prevent further decline in serum chloride levels in patients with HF [25]. No studies have explored the effects of treatment with ARBs, ACEis, or beta-blockers on chloride homeostasis. The majority of subjects in our study with HfrEF were treated with other GDMTs alongside SGLT2is, so we believe that the possible influence of concomitant therapy on our results is small. However, SGLT2i therapy in HF is usually used in addition to other medications that primarily induce diuresis, such as loop diuretics, which do impact the chloride concentration [11,15]. The need for diuretics is not constant in patients with HF. An increase in the dosage of diuretics can be related to worsening HF as well as the development of diuretic resistance or reduced kidney function, while a decrease in the dosage of diuretics can be related to compensation or cardiac remodeling with GDMT [31]. Although it seems reasonable that patients taking both a diuretic and an SGLT2i would need an alteration their loop diuretic dose, to date there is no guidance regarding which patients are most likely to require diuretic alterations, or to what degree their doses should be altered. The dose of loop diuretics in a real-world setting is usually determined by physician preference. We investigated the impact of loop diuretics (specifically, furosemide) at different dosages (low, medium, or high) on serum chloride concentration in the follow-up period. The low furosemide dose was 20–40 mg/day, the medium dose was 40–80 mg/day, and the high dose was more than 80 mg/day. Low and medium doses of furosemide had a significant effect on the increase in chloride levels after 6 and 12 months of follow-up, respectively. One of the limitations of our observational study is that we do not have data on the extent to which the diuretic doses changed during the follow-up, that is, how they were adjusted; we only have data on what the doses were after 6 months and 12 months of follow-up. Unfortunately, we do not know whether they had changed in the meantime. We can only assume that they were the same in patients who were not hospitalized or did not visit the emergency department due to worsening of HF. Our study found that patients who were treated with higher doses of loop diuretics had lower chloride concentrations during follow-up. We cannot know whether this was the result of their clinical condition, i.e., whether the leading physicians assessed that the dose of diuretics could not be de-escalated during follow-up, or whether the physicians were not inclined to do so due to clinical inertia.

Furthermore, we evaluated the correlation between chloride concentrations, NT-proBNP concentration, and functional status of the patient. NT-proBNP is the key biochemical marker in the diagnosis and monitoring of HF patients and in guiding therapy. Daubert et al. [32] showed that achieving target NT-proBNP concentrations of less than 1000 pg/mL by 12 months is associated with significant reverse remodeling and improved clinical outcomes in HFrEF patients. It possibly plays a very important role as a potential “left ventricular remodeling surrogate”. Our results show that the increase in the chloride concentration was significantly correlated with lower concentrations of NT-proBNP and with a better functional status of the patient according to the NYHA classification, both at 6 and 12 months of follow-up, indicating a good therapy response. The highest chloride concentrations were recorded in the lower NYHA groups, both after 6 and 12 months of follow-up. These findings are consistent with the results of previous studies, in which it was shown that low chloride concentrations were associated with worsening HF and an increased risk of mortality in patients with HFrEF, but also in patients with HFpEF [7].

We also examined the association of some other patient-specific factors—such as age, gender, smoking history, and BMI—with NT-proBNP concentration. Older age itself represents the risk of additional comorbidities and is a known risk factor for deteriorating heart function, so older age is associated with an increase in NT-proBNP both after 6 and 12 months. Smoking history, also a risk factor for additional cardiovascular comorbidities and for deteriorating heart function, is significantly associated with NT-proBNP concentration. Previous studies have suggested that cigarette smoking increases cardiac overload and increases the serum concentration of NT-proBNP [33]. In our study, the results were consistent with previous findings, but significant results were obtained only after 12 months of follow-up. Obesity is a risk factor for the development of HF, especially HFpEF [34]. Unlike age and smoking, BMI is inversely correlated with NT-proBNP concentration. This has been observed by numerous earlier studies, due to impaired NP production in obese patients [35,36]. The study by Bayes-Genis [37] showed that, in patients with and without acute HF, the NT-proBNP concentrations were relatively lower in overweight (25–29.9 kg/m^2^) and obese (more than 30 kg/m^2^) patients with acute dyspnea. However, the precise cut-off points when assessing patients with both HF and high BMI are unknown. Clinically, the risk for lower NP concentrations is observed in those with BMI ≥ 30 kg/m^2^. To optimize diagnostic accuracy, lowering the established cut-off NT-proBNP concentrations by up to 50% in obese patients seems reasonable. A very low BNP cut-off concentration (less than 50 pg/mL) should be used to rule out HF [38]. Importantly, the NT-proBNP concentration retains its diagnostic and prognostic capacity across all BMI categories. However, additional studies will be needed to determine in more detail the cut-off concentration of NT-proBNPa that is indicative of the diagnosis of HF in different groups of obese patients, taking into account associated comorbidities such as atrial fibrillation, and bearing in mind the importance of a patient-tailored approach. In our study, gender did not affect NT-pro BNP concentrations. Previous studies showed that women tend to have higher levels of NT-proBNP; however, these results should be interpreted with caution given the differences between women and men, including the epidemiology, etiology, pathogenesis, risk factors, and prognosis of HF [39]. Our study included less than 30% women and included patients with HF in the whole spectrum of ejection fraction, which was probably the reason for this result.

Two recent studies have shown that it is also essential to analyze the Na/Cl ratio in HF patients, and that the observed increase in the Na/Cl ratio can be attributed to a modest yet significant rise in serum sodium, primarily accompanied by a notable decrease in serum chloride concentration [21,22,40]. Both studies included patients with acute HF and critically ill patients and were performed before the era of SGLT2 inhibitors in heart failure. The Na/Cl ratio was determined only during hospitalization. Although our primary goal was to examine the effects of SGLT2is on chloride concentrations, we could not ignore the decades of the ”reign of sodium”, especially after learning about the influence of the Na/Cl ratio on the outcomes of patients with acute HF. Therefore, we examined the Na/Cl ratios the baseline and after 6 and 12 months. As far as we know, this is the first study to analyze the dynamics of the Na/Cl ratio after 12 months of follow-up in heart failure patients with SGLT2is included in their therapy. However, there were no statistically significant differences in Na/Cl ratio between different timepoints. A possible reason for such a finding is the effect of SGLT2is, which we found to increase the concentration of chloride. Although their effect on sodium concentration is also not fully understood, research supports the theory that SGLT2is increase sodium concentration. Yeoh et al. [26,41] performed a post hoc analysis of the DAPA-HF trial with a focus on serum sodium [29]. According to their observations, there appears to be a biphasic effect of dapagliflozin on serum sodium. When exposed to dapagliflozin, the sodium concentration initially lowers more than with placebo, but it then shows an increasing trend that rises above both baseline and the placebo group by 8 months. This phenomenon is likely caused by a combination of natriuretics in the early stages and osmotic effects of SGLT2 inhibitors in their later stages of action. We hypothesize that the collective action of SGLT2is on sodium and chloride ultimately does not disturb their ratio to a significant extent, which might be the reason why the Na/Cl ratio in our population did not differ significantly at different timepoints. Further studies will certainly be needed to test our thesis. We also tested the correlation between Na/Cl ratio and NT-proBNP concentration, finding that higher values of Na/Cl ratio are independent predictors of higher NT-proBNP concentration at all timepoints. As expected, there was a very strong correlation between chloride concentration and Na/Cl ratio at all timepoints.

Finally, we evaluated the relationship between serum chloride concentrations and the odds of hospital admission or emergency department visits due to HF worsening in the follow-up. Our results show that a higher initial chloride concentration was significantly associated with decreased odds of hospital admission due to worsening of HF. Although our results indicate the possibility that hypochloremia could be a modifiable risk factor, we must emphasize that most of the information about the relationship between chloride concentrations and HF outcomes originates from post hoc analysis of studies in which chloride was not a primary or secondary endpoint. As such, further prospective clinical trials are needed to elucidate whether hypochloremia is indeed a modifiable risk factor or just a marker of the severity of HF disease and ominous signs of a poor outcome.

## 5. Conclusions

Despite the growing body of evidence from post hoc analyses of randomized clinical trials, as well as data from observational studies on the influence of chloride concentrations on outcomes in patients with HF, it appears that the determination of chloride concentrations in patients with HF has not yet become routine in the follow-up of patients with HF. Our results suggest that hypochloremia could be a potentially modifiable risk factor, given the influence of SGLT2is on chloride concentration, but also an ominous sign of a poor outcome in patients with HF. We believe that the determination of chloride concentrations should become routine in the monitoring of patients with HF.

## 6. Limitations

The present study has some limitations. First, it is based on data from an observational registry. Due to the character of the registry (eligibility depending on initiation of SGLT2i therapy), patients with contraindications for SGLT2is were omitted (e.g., type 1 diabetes, frequent urinary infections). Second, this observational registry was not followed up with a detailed study protocol, and due to its nature, the intervention allocation, i.e., SGLT2i initiation, was entirely dependent on the clinician’s decision. Third, since this was a single-center study, the number of patients was limited. Finally, data regarding the extent to which diuretic doses were changed during the follow-up are not available, which is an important limitation. We do not have information on how exactly the clinicians assessed the degree of congestion in a particular patient, or on what they based their decision to change or not change the dose of diuretics.

## Figures and Tables

**Table 1:** Baseline demographic characteristics of the study population. Data are presented as medians with IQRs for continuous variables, and as counts with proportions for categorical variables; *n* = 241.

Category	*n*	Percentage
Gender		
Male	172	71.37
Female	69	28.63
NYHA status		
1	14	5.81
2	133	55.19
3	91	37.76
4	3	1.24
Age (C, IQR)	67 (58–72)	
BMI (C, IQR)	28.8 (25.7–32.2)	
Heart rate—admission (C, IQR)	82 (72–98)	
Heart rate—discharge (C, IQR)	75 (66–84)	
Atrial fibrillation		
AF paroxysmal	30	12.45
AF persistent	32	13.28
AF Permanent	35	14.52
Sinus rhythm	144	59.75
Arterial hypertension		
No	36	14.94
Yes	205	85.06
Diabetes mellitus type 2		
No	60	24.9
Yes	74	30.71
Yes—de novo	26	10.79
Pre-diabetes	81	33.61
Smoking		
No	141	58.51
Yes	100	41.49
Coronary artery disease		
No	108	44.81
Yes	132	54.77
Hyperlipidemia		
No	62	25.73
Yes	179	74.27
Cerebrovascular disease—stroke/TIA		
No	217	90.04
Yes	24	9.96
Peripheral arterial disease		
No	188	78.01
Yes	53	21.99
COPD		
No	212	87.97
Yes	29	12.03
Laboratory findings (C, IQR)		
Urea (mmol/L)	7.6 (6–9.72)	
Creatinine (μmol/L)	90 (75–113)	
eGFR (mL/min/1.73 m^2^)	69.3 (53.1–89)	
MCV (fl)	90.05 (86.5–93.43)	
Glucose (mmol/L)	6.6 (5.6–8.5)	
CRP (mg/L)	5.1 (2.23–11.8)	
Albumin (g/L)	41 (39–41)	
NT-proBNP	2020.5 (998.5–5126.75)	
Cholesterol (mmol/L)	4.4 (3.7–5.6)	
LDL (mmol/L)	2.7 (2–3.6)	
HDL (mmol/L)	1.2 (1–1.4)	
Triglyceride (mmol/L)	1.2 (1–1.7)	
HbA1c (%)	6.1 (5.7–6.7)	
Potassium (mmol/L))	4.3 (4–4.6)	
Sodium (mmol/L)	139 (137–141)	
Chloride (mmol/L)	102 (100–104)	

**Table 2:** Concomitant therapy before introduction of SGLT2is. Data are presented as medians with IQRs for continuous variables, and as counts with proportions for categorical variables; *n* = 241.

Concomitant Therapy	*n*	Percentage
ACE inhibitors		
No	118	48.96
Yes	123	51.04
ACE inhibitors—medication		
Ramipril	40	16.6
Perindopril	80	33.2
Lizinopril	1	0.41
Zofenopril	2	0.82
Angiotensin receptor blocker		
No	226	93.78
Yes	15	6.64
Angiotensin receptor blocker—medication		
Valsartan	13	5.39
Telmisartan	2	0.83
Beta-blockers		
No	23	8.7
Yes	218	91.3
Beta-blockers—medication		
Bisoprolol	165	68.46
Metoprolol	20	8.3
Carvedilol	21	8.71
Nebivolol	12	4.98
Mineralocorticoid receptor antagonist		
No	41	17.01
Yes	200	82.99
MRA—medication		
Eplerenone	199	82.57
Spironolactone	1	0.41
Diuretics		
No	68	28.22
Yes	173	71.78
Diuretics—medication		
Furosemide	151	62.66
Indapamide	7	2.9
Torasimide	10	4.15
Hydrochlorothiazide	4	1.66
Chlortalidone	1	0.41
ARNi		
No	161	66.8
Yes	80	33.2
ARNI—dose		
24/26mg BID	24	9.96
49/51 BID	29	12.03
97/103 BID	27	11.2
SGLT2 inhibitors		
Dapagliflozine	121	50.21
Empagliflozine	120	49.79
SGLT2 inhibitors—dose		
10 mg	216	89.63
12.5 mg	25	10.37
GLP-1		
No	212	87.97
Yes	29	12.03

**Table 3:** Chloride concentrations at baseline, 6 months, and 12 months. Data are presented as medians with IQRs for continuous variables.

Timepoint	Median Chloride Concentration	Interquartile Range
Baseline	102	100–104
6 Months	103	101–105
12 Months	103	101–105

**Table 4:** Na/Cl ratio at baseline, 6 months, and 12 months. Data are presented as medians with IQRs for continuous variables.

Timepoint	Median Na/Cl	Interquartile Range
Baseline	1.36	1.33–1.38
6 Months	1.35	1.32–1.38
12 Months	1.35	1.33–1.37

**Table 5:** Chloride concentration and Na/Cl ratio at baseline, 6 months, and 12 months for different HF groups. Data are presented as medians with IQRs for continuous variables. * Statistically significant value calculated using the Kruskal–Wallis test with post hoc Mann–Whitney U test for continuous variables, and using the χ^2^ test for categorical variables.

Variable	HFpEF (*n* = 56)	HFmrEF (*n* = 34)	HFrEF (*n* = 151)	*p*-Value
Chloride concentration				
Baseline	102 (100–104)	103 (100–105)	102 (100–104)	0.748
6 Months	103 (101–104)	104 (102–106)	103 (101–105)	0.151
12 Months	103 (102–104)	104 (102–105)	103 (101–105)	0.381
Na/Cl ratio				
Baseline	1.36 (1.34–1.38)	1.35 (1.32–1.37)	1.36 (1.33–1.39)	0.240
6 Months	1.35 (1.32–1.36)	1.31 (1.30–1.36)	1.36 (1.32–1.38)	0.018 *
12 Months	1.36 (1.34–1.37)	1.34 (1.32–1.37)	1.35 (1.33–1.38)	0.169

**Table 6:** NT-proBNP values at baseline, 6 months, and 12 months according to HF phenotype.

		Median	Minimum	Maximum	25th Percentile	75th Percentile
NT-proBNP at baseline	HFpEF	1430	137	26,735	597	3709
	HFmrEF	1761	198	32,127	987	4397
	HFrEF	3083	129	35,000	1264	6407
NT-proBNP at 6 months	HFpEF	643	45	8483	299.75	1822.25
	HFmrEF	849.5	32	10,121	301.5	1066
	HFrEF	833	72	35,000	493.75	1882
NT-proBNP at 12 months	HFpEF	451	59	7192	272	1106
	HFmrEF	592	20	12,555	165.5	927
	HFrEF	651	43	35,000	325.5	1225.5

**Table 7:** Multivariate regression analysis with Cl concentration as a covariate. Linear relationships between variables and natural logarithm of NT-proBNP level were tested at baseline, 6 months, and 12 months. * Statistically significant result. N/A: not applicable—used for non-significant variables from univariate analysis. Data are given as parameter estimates with 95% CIs (confidence intervals).

NT-proBNP at Baseline				
	Univariate analysis		Multivariate analysis	
Variable	Parameter estimate (95% CI)	*p*-Value	Parameter estimate (95% CI)	*p*-Value
Cl at baseline	−0.05 (−0.10; −0.01)	0.014 *	−0.05 (−0.09; −0.01)	0.029 *
Gender (F vs. M)	0.17 (−0.16; 0.50)	0.312	N/A	N/A
Age	0.03 (0.02; 0.04)	<0.001 *	0.02 (0.01; 0.03)	<0.001 *
BMI	−0.03 (−0.06; −0.01)	0.013 *	−0.02 (−0.05; 0.00)	0.074
Smoking (yes vs. no)	0.14 (−0.16; 0.45)	0.351	N/A	N/A
			R^2^ = 0.114	
NT-proBNP at 6 Months				
	Univariate analysis		Multivariate analysis	
Variable	Parameter estimate (95% CI)	*p*-Value	Parameter estimate (95% CI)	*p*-Value
Cl at 6 months	−0.07 (−0.12; −0.03)	0.03 *	−0.07 (−0.11; −0.03)	0.003 *
Gender (F vs. M)	0.28 (−0.03; 0.60)	0.079	N/A	N/A
Age	0.04 (0.03; 0.05)	<0.001 *	0.04 (0.03; 0.05)	<0.001 *
BMI	−0.05 (−0.07; −0.02)	0.01 *	−0.03 (−0.06; −0.01)	0.008 *
Smoking (yes vs. no)	−0.07 (−0.36; 0.23)	0.649	N/A	N/A
			R^2^ = 0.216	
NT-proBNP at 12 Months				
	Univariate analysis		Multivariate analysis	
Variable	Parameter estimate (95% CI)	*p*-Value	Parameter estimate (95% CI)	*p*-Value
Cl at 12 months	−0.10 (−0.15; −0.05)	<0.001 *	−0.09 (−0.14; −0.05)	<0.001 *
Gender (F vs. M)	0.28 (−0.08; 0.63)	0.125	N/A	N/A
Age	0.04 (0.03; 0.05)	<0.001 *	0.04 (0.02; 0.05)	<0.001 *
BMI	−0.05 (−0.08; −0.02)	<0.001 *	−0.03 (−0.05; −0.01)	0.014 *
Smoking (yes vs. no)	0.00 (−0.32; 0.32)	0.997	N/A	N/A
			R^2^ = 0.253	

**Table 8:** Multivariate regression analysis with Na/Cl ratio as a covariate. Linear relationships between variables and natural logarithm of NT-proBNP concentration were tested at baseline, 6 months, and 12 months. * Statistically significant result. N/A: not applicable—used for non-significant variables from univariate analysis. Data are given as parameter estimates with 95% CIs (confidence intervals).

NT-proBNP at Baseline				
	Univariate analysis		Multivariate analysis	
Variable	Parameter estimate (95% CI)	*p*-Value	Parameter estimate (95% CI)	*p*-Value
Na/Cl at baseline	5.84 (2.42; 9.25)	<0.001 *	4.97 (1.66; 8.28)	0.003 *
Gender (F vs. M)	0.17 (−0.16; 0.50)	0.312	N/A	N/A
Age	0.03 (0.02; 0.04)	<0.001 *	0.03 (0.01; 0.04)	<0.001 *
BMI	−0.03 (−0.06; −0.01)	0.013 *	−0.03 (−0.05; 0.00)	0.056
Smoking (yes vs. no)	0.14 (−0.16; 0.45)	0.351	N/A	N/A
			R^2^ = 0.128	
NT-proBNP at 6 Months				
	Univariate analysis		Multivariate analysis	
Variable	Parameter estimate (95% CI)	*p*-Value	Parameter estimate (95% CI)	*p*-Value
Na/Cl at 6 months	3.46 (0.21; 6.72)	0.037 *	3.71 (0.80; 6.61)	<0.013 *
Gender (F vs. M)	0.28 (−0.03; 0.60)	0.079	N/A	N/A
Age	0.04 (0.03; 0.05)	<0.001 *	0.04 (0.03; 0.05)	<0.001 *
BMI	−0.05 (−0.07; −0.02)	0.01 *	−0.03 (−0.06; −0.01)	0.015 *
Smoking (yes vs. no)	−0.07 (−0.36; 0.23)	0.649	N/A	N/A
			R^2^ = 0.231	
NT-proBNP at 12 Months				
	Univariate analysis		Multivariate analysis	
Variable	Parameter estimate (95% CI)	*p*-Value	Parameter estimate (95% CI)	*p*-Value
Na/Cl at 12 months	7.31 (3.91; 10.71)	<0.001 *	6.71 (3.62; 9.79)	<0.001 *
Gender (F vs. M)	0.28 (−0.08; 0.63)	0.125	N/A	N/A
Age	0.04 (0.03; 0.05)	<0.001 *	0.04 (0.03; 0.05)	<0.001 *
BMI	−0.05 (−0.08; −0.02)	<0.001 *	−0.03 (−0.05; −0.01)	0.016 *
Smoking (yes vs. no)	0.00 (−0.32; 0.32)	0.997	N/A	N/A
			R^2^ = 0.247	

**Table 9:** Multivariate binary regression for increase in chloride concentration at 6 and 12 months. * Statistically significant result. N/A: not applicable—used for non-significant variables from univariate analysis. Data are given as parameter estimates with 95% CIs (confidence intervals).

Increase in Chloride Concentration at 6 Months				
	Univariate analysis		Multivariate analysis	
Variable	Parameter estimate (95% CI)	*p*-Value	Parameter estimate (95% CI)	*p*-Value
CAD (yes vs. no)	−0.35 (−0.86; 1.16)	0.183	N/A	N/A
DM (yes vs. no)	0.30 (−0.29; 0.89)	0.315	N/A	N/A
LVEF at baseline	−0.01 (−0.03; 0.12)	0.432	N/A	N/A
NT-proBNP at baseline	0.00 (0.00; 0.00)	0.907	N/A	N/A
Furosemide in therapy (yes vs. no)	0.49 (−0.03; 1.00)	0.065	N/A	N/A
Furosemide dose			N/A	N/A
Large vs. no	0.27 (−0.35; 0.89)	0.392	N/A	N/A
Medium vs. no	0.83 (−0.10; 1.76)	0.800	N/A	N/A
Small vs. no	1.12 (0.11; 2.13)	0.030 *	1.12 (0.11; 2.13)	0.030 *
Increase in Chloride Concentration at 12 Months				
	Univariate analysis		Multivariate analysis	
Variable	Parameter estimate (95% CI)	*p*-Value	Parameter estimate (95% CI)	*p*-Value
CAD (yes vs. no)	−0.21 (−0.72; 0.30)	0.416	N/A	N/A
DM (yes vs. no)	0.18 (−0.41; 0.77)	0.544	N/A	N/A
LVEF at baseline	−0.01 (−0.03; 0.0.01)	0.550	N/A	N/A
NT-proBNP at baseline	−0.01 (−0.04; 0.03)	0.628	N/A	N/A
Furosemide in therapy (yes vs. no)	0.25 (−0.28; 0.77)	0.354	N/A	N/A
Furosemide dose			N/A	N/A
Large vs. no	−0.10 (−0.72; 0.52)	0.753	N/A	N/A
Medium vs. no	1.06 (0.01; 2.12)	0.049 *	1.06 (0.01; 2.12)	0.049 *
Small vs. no	0.52 (−0.46; 1.50)	0.297	N/A	N/A

**Table 10:** Multivariate ordinal regression for number of hospital admissions due to worsening heart failure during follow-up. * Statistically significant result. N/A: not applicable—used for non-significant variables from univariate analysis. Data are given as parameter estimates with 95% CIs (confidence intervals).

Number of Hospitalizations at Follow-Up (*n* = 14)				
	Univariate Analysis		Multivariate Analysis	
Variable	Parameter Estimate (95% CI)	*p*-Value	Parameter Estimate (95% CI)	*p*-Value
CAD (yes vs. no)	−0.16 (−0.32; 0.64)	0.517	N/A	N/A
DM (yes vs. no)	0.25 (−0.29; 0.80)	0.365	N/A	N/A
LVEF at baseline	−0.02 (−0.04; 0.00)	0.045 *	−0.02 (−0.04; 0.00)	0.054
NT-proBNP at baseline	0.00 (0.00; 0.00)	0.602	N/A	N/A
Cl at baseline	−0.08 (−0.15; −0.01)	0.028 *	−0.08 (−0.15; −0.01)	0.033 *
Na/Cl at baseline	4.18 (−1.38; 9.84)	0.143	N/A	N/A

**Table 11:** Chloride concentrations at 6 months and 12 months for different NYHA groups. Data are presented as medians with IQRs for continuous variables. The Kruskal–Wallis test was used. * Statistically significant results.

	NYHA 1	NYHA 2	NYHA 3	*p*-Value
Cl at 6 months	104 (102–105)	103 (101–105)	101 (98–102)	<0.013 * 1 vs. 2 0.187 1 vs. 3 0.024 * 2 vs. 3 0.125
Cl at 12 months	103 (102–105)	103 (100–104)	100 (96–103)	<0.001 * 1 vs. 2 0.031 * 1 vs. 3 0.003 * 2 vs. 3 0.072

## Data Availability

Data will be available upon request.
